# Brain injury, endothelial injury and inflammatory markers are elevated and express sex-specific alterations after COVID-19

**DOI:** 10.1186/s12974-021-02323-8

**Published:** 2021-11-27

**Authors:** Jude Savarraj, Eun S. Park, Gabriela D. Colpo, Sarah N. Hinds, Diego Morales, Hilda Ahnstedt, Atzhiry S. Paz, Andres Assing, Fudong Liu, Shivanki Juneja, Eunhee Kim, Sung-min Cho, Aaron M. Gusdon, Pramod Dash, Louise D. McCullough, H. Alex Choi

**Affiliations:** 1 Departent of Neurosurgery, McGovern Medical School, The University of Texas Health Science Center at Houston, 6431 Fannin St, Houston, TX 77030 USA; 2 Department of Neurobiology, McGovern Medical School, The University of Texas Health Science Center at Houston, Houston, TX 77030 USA; 3 Johns Hopkins University School of Medicine, Baltimore, MD 21205 USA; 4 Department of Neurology, McGovern Medical School, The University of Texas Health Science Center at Houston, Houston, TX 77030 USA

**Keywords:** Brain injury, COVID-19, SARS-CoV-2, Inflammation, Endothelial injury, Sex differences

## Abstract

**Objective:**

Although COVID-19 is a respiratory disease, all organs can be affected including the brain. To date, specific investigations of brain injury markers (BIM) and endothelial injury markers (EIM) have been limited. Additionally, a male bias in disease severity and mortality after COVID-19 is evident globally. Sex differences in the immune response to COVID-19 may mediate this disparity. We investigated BIM, EIM and inflammatory cytokine/chemokine (CC) levels after COVID-19 and in across sexes.

**Methods:**

Plasma samples from 57 subjects at < 48 h of COVID-19 hospitalization, and 20 matched controls were interrogated for the levels of six BIMs—including GFAP, S100B, Syndecan-1, UCHLI, MAP2 and NSE, two EIMs—including sICAM1 and sVCAM1. Additionally, several cytokines/chemokines were analyzed by multiplex. Statistical and bioinformatics methods were used to measure differences in the marker profiles across (a) COVID-19 vs. controls and (b) men vs. women.

**Results:**

Three BIMs: MAP2, NSE and S100B, two EIMs: sICAM1 and sVCAM1 and seven CCs: GRO IL10, sCD40L, IP10, IL1Ra, MCP1 and TNFα were significantly (*p* < 0.05) elevated in the COVID-19 cohort compared to controls. Bioinformatics analysis reveal a stronger positive association between BIM/CC/EIMs in the COVID-19 cohort. Analysis across sex revealed that several BIMs and CCs including NSE, IL10, IL15 and IL8 were significantly (*p* < 0.05) higher in men compared to women. Men also expressed a more robust BIM/ EIM/CC association profile compared to women.

**Conclusion:**

The acute elevation of BIMs, CCs, and EIMs and the robust associations among them at COVID-19 hospitalization are suggestive of brain and endothelial injury. Higher BIM and inflammatory markers in men additionally suggest that men are more susceptible to the risk compared to women.

**Supplementary Information:**

The online version contains supplementary material available at 10.1186/s12974-021-02323-8.

## Introduction

Neurologic complications after COVID-19 have been reported in both the acute and chronic period [1, 2]. Neurologic manifestations including ischemic and hemorrhagic stroke have been reported after COVID-19. But neurologic manifestations without clear etiology including encephalopathy, pain, chronic fatigue and cognitive dysfunction have also been reported demonstrating the wide impact of COVID-19 on the nervous system [3–8]. Human coronaviruses (HCoV) are neuro-invasive and neurotropic by nature and may contribute to both short- and long-term neurological disorders [9–11]. SARS-CoV (of the SARS epidemic in early 2000s) has been shown to affect the CNS [12] and viral strains have been identified in post-mortem human brain tissue [12, 13]. Our understanding of the effect of COVID-19 on brain injury is only beginning. COVID-19 triggers multiple pathological mechanisms—including an acute inflammatory response and endotheliopathy—and the relationship of these mechanisms with brain injury is unclear.

Sex differences in COVID-19 are prominent, with men having more severe disease, with higher hospitalization and mortality rates, despite similar rates of infection—an effect seen globally [14, 15]. Sex differences in immune response may underlie the increase in mortality seen in men during the acute disease course [16, 17]. It is uncertain whether the increase in peripheral inflammation seen in accompanied by an increase in brain injury markers as well.

We investigated brain injury markers (BIMs), endothelial injury markers (EIMs) and inflammatory markers in COVID-19 patients in the acute phase (< 48 h) after hospitalization. Several BIMs have been reported as surrogate markers of neuronal and astrocytic injury in non-neurological diseases like HIV [18–20], cardiac arrest [21–23] and sepsis [24]. Among them, we measured six validated BIMs including glial fibrillary acidic protein (GFAP), neuron-specific enolase (NSE), S100B, ubiquitin carboxyl-terminal hydrolase isozyme L1 (UCHL1), Syndecan-1 and microtubule-associated protein 2 (MAP 2) in plasma drawn from hospitalized COVID-19 subjects and matched controls. In addition to BIMs, we measured two EIMs [including *Intercellular Adhesion Molecule 1* (ICAM-1) and *Vascular Cell Adhesion Molecule 1* (VCAM-1)] and cytokine/chemokine markers (CCs) of systemic inflammation to investigate if endothelial injury and inflammation are associated with BIMs. The levels of the markers were measured by ELISA and multiplex assays, and the association between COVID-19 and controls were characterized using statistical and bioinformatics approaches. The differences in the levels of markers between men and women were investigated.

## Methods

### Study population and patient inclusion and exclusion criteria

This is a prospective study of COVID-19 patients admitted and hospitalized at the Memorial Herman Hospital System in Houston, Texas, USA. Inclusion criteria were laboratory-confirmed SARS-CoV-2 infection by real-time polymerase chain reaction, written informed consent from the patient or surrogate, and age ≥ 18 years. Exclusion criteria were inability to complete long-term follow-up, severe functional disabilities before hospital admission for COVID-19 [defined by pre-admission modified Rankin Score (mRS) [25] > 1], history of pulmonary complications (including resection and transplant), pre-existing systemic diseases which would impact long-term outcomes (including stroke, myocardial infarction, pulmonary disease requiring home oxygen, chronic renal failure necessitating hemodialysis and malignancy), documented neurologic and psychiatric disorders, prisoners and pregnant women. Patients were categorized as mild (nasal cannula with < 5 L of O_2_ and < 5 days of hospitalization), moderate (nasal cannula with > 5 L of O_2_ or heat high-flow cannula and > 5 days of hospitalization) and severe (on ventilator or expired). Plasma samples from 24 non-neurological subjects who were enrolled at the *UT Physician Cardiology* clinic were used as controls. The controls were matched to the COVID-19 cohort for age, sex and co-morbidities (including diabetes and hypertension). This study was approved by the ‘Institutional Review Board’ (IRB No: HSC –MH-17-0452) at The University of Texas Health Science Center at Houston, Houston, Texas.

### Samples

Blood was drawn by venipuncture, collected into sterile vacutainers, and immediately placed on ice. For processing of plasma, the tubes were centrifuged at 1200×*g* for 10 min at 4 °C followed by a second centrifugation at 10,000×*g* for 10 min at 4 °C to generate platelet-poor plasma. Plasma were then aliquoted and stored at − 80 °C until the samples were analyzed.

### ELISA and multiplex analysis

BIMs were measured using the following ELISA kits: GFAP (Cat No: NS830, Sigma-Aldrich), NSE (Cat No: 420-85, IBL America), S100B (Cat No: EZHS100B-33K, MilliporeSigma), UCH-L1 (EH475RB, ThermoFisher), syndecan-1(RAB0736-1KT, MilliporeSigma) and MAP2 (EKU05950, BIOMATIK). The EIMs (including ICAM-1 and VCAM-1) were measured using a premixed multiplex assay (Cat. #HCVD2MAG-67K, MilliporeSigma). Systemic levels of CCs were measured using a 38-plex premixed immunological multiplex assay (HCYTMAG-60K-PX38, MilliporeSigma, Billerica, MA). CCs included CD40L, EGF, Eotaxin/CCL11, FGF-2, Flt-3 ligand, Fractalkine, G-CSF, GM-CSF, GRO, IFN-α2, IFN-γ, IL-1α, IL-1β, IL-1ra, IL-2, IL-3, IL-4, IL-5, IL-6, IL-7, IL-8, IL-9, IL-10, IL-12 (p40), IL-12 (p70), IL-13, IL-15, IL-17A, IP-10, MCP-1, MCP-3, MDC (CCL22), MIP-1α, MIP-1β, TGF-α, TNF-α, TNF-β and VEGF. All experiments were performed according to manufacturer’s protocol. The levels of all markers are reported as pg/ml.

### Statistical analysis

Descriptive statistics were calculated for demographic variables and protein levels in control and COVID-19 subjects. To describe differences in demographics, *χ*^2^-test, Fisher’s exact test, Student’s *t*-test, and the Mann–Whitney *U* test were used where appropriate. The Mann–Whitney U test was used to test for differences in protein levels across different groups A *p*-value of ≤ 0.05 was considered statistically significant (two-tailed). The levels of the markers are reported as mean ± standard error (SE). All statistical analyses were performed using open-source software packages in R (v3.1.3).

### Bioinformatics and chord diagrams

To elucidate the relationship between the BIMs, EIMs and CCs, we employed the chord diagram technique that is useful in visualizing relationships in multi-dimensional data. A chord diagram is a graphical method that is typically used to display inter-relationships between large numbers of variables. In this study, the variables are the markers that were investigated (the CCs, BIMs and the EIMs) and the relationship between them is the Pearson’s correlation coefficient (PCC). Each marker distribution was first normalized via Box–Cox transformations [26] and the PCCs were calculated (R v3.1.3) for each pair of the markers. This resulted in a large correlation matrix with each entry in the matrix representing a PCC between corresponding markers. We retained only the significant (*p* < 0.05) PCCs and constructed the chord diagrams. In the chord diagram, we arranged each marker sub-type (i.e., CCs, BIMs and EIMs) as an outer sector that were color-coded (green for CCs, red for BIMs and dark blue for EIMs) to maintain visualization consistent with other figures presented. The variables (the CCs, BIMs and the EIMs) were represented by the inner-sectors and the length of the inner-sectors was mapped to the number of significant PCCs that the corresponding variable had with other variables in the dataset. A longer inner-sector denotes that the corresponding variable had a high number of PCCS with other markers. The random-skewers method [27] was used to test for statistical differences across the chord diagrams. The R package *circlize* [28] was used to construct the chord diagrams.

### Modified Rankin assessment

The functional status of the patient was quantified using the modified Rankin’s scale (mRS) which is a 0–6 point grading scale of the patient’s functional status [29]. ‘0’: no deficits, ‘1’: presence of symptoms but no significant disability, ‘2’: a slight disability but independent, ‘3’: moderate disability requiring some help but able to walk without assistance, ‘4’: a moderately severe disability and unable to walk and attend to bodily needs without assistance, ‘5’: a severe disability where the patient requires constant nursing care and attention and, ‘6’: being dead. For the purposes of this study, we dichotomized patients into two groups; good (mRS ≤ 3) and poor (mRS ≥ 4) outcomes. The mRS at discharge was assessed.

## Results

### Patient characteristics

Samples from 57 subjects and 20 control subjects were included in the study. Demographics and clinical outcomes of the COVID-19 subjects and controls are presented (Table 1). There were no significant differences in age and sex distribution between the two groups. The COVID-19 cohort had a significantly more Hispanic subjects compared to the control (56% vs. 10%, *p* < 0.01). There were no differences in the history of hypertension, diabetes and coronary heart disease between the two groups. However, the COVID-19 group had more obese subjects compared to controls (49% vs. 20%, *p* = 0.03).

**Table 1:** Demographics and past medical history

Variables	Control	COVID-19	*p*
	*N* = 20	*N* = 57	
Demographics			
Age (mean, sd)	61 (15)	53 (18)	0.07
Sex, female (*n*, %)	11 (55)	24 (42)	0.4
Race (*n*, %)			
White	16 (80)	35 (61)	0.17
African-American	3 (15)	15 (26)	0.37
Others	1 (5)	7 (12)	0.67
Ethnicity, Hispanic (*n*, %)	2 (10)	32 (56)	**< 0.01**
Past medical history			
Hypertension (*n*, %)	5 (25)	24 (42)	0.28
Diabetes (*n*, %)	4 (20)	24 (42)	0.16
CAD (*n*, %)	5 (25)	7 (12)	0.16
Obesity (*n*, %)	4 (20)	28 (49)	**0.03**

*p*-values < 0.05 are shown in bold

*SD* standard deviation, *CAD* coronary artery disease

### BIMs after COVID-19 hospitalization

**Fig. 1:**
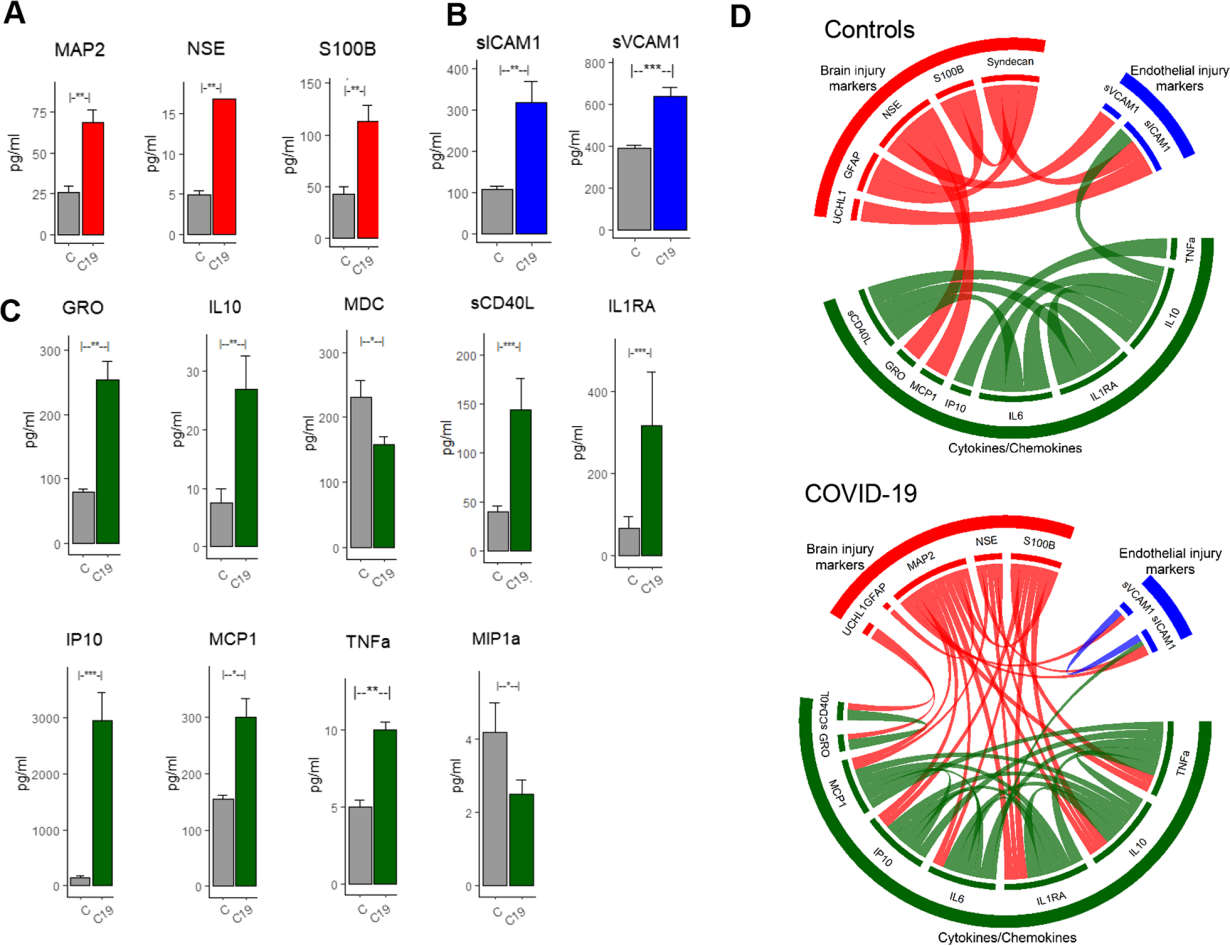
Difference in BIM, EIM and CC profile across COVID-19 subjects and matched controls. **A** BIMs including MAP2, NSE and S100B were significantly (*p* < 0.05) higher immediately after COVID-19 hospitalization. **B** sICAM1 and SVCAM1 levels were significantly higher after COVID-19 hospitalization. **C** Several inflammatory markers including GRO, IL10, sCD40L, IL1RA, IP10, MCP1 and TNFa were significantly higher after COVID-19. MDC and MIP1a were significantly lower after COVID-19. **D** Chord diagram reveals significant (*p* < 0.05, Fishers’ test) differences in interactions between the BIM, EM, and CCs across COVID-19 and control groups

At < 48 h hospitalization for of COVID-19, the mean levels of MAP2 (68 ± 7.5 vs. 26 ± 3.9, pg/ml, *p* < 0.01), NSE (16.9 ± 3.2 vs. 4.9 ± 0.59, pg/ml, *p* < 0.01) and S100B (113 ± 16 vs. 43 ± 7, pg/ml, *p* < 0.01) were significantly higher compared to controls (Fig. 1A). In the COVID-19 cohort, the average levels of MAP2, NSE and S100B were 160%, 245% and 162% higher than controls.

### EIMs and inflammatory CCs after COVID-19 hospitalization

At the acute phase (< 48 h), the levels of the EIMs and most CCs were significantly higher in the COVID-19 cohort compared to controls. EIMs including sICAM1 (317 ± 51 vs. 108 ± 7.5, pg/ml, *p* < 0.001) and sVCAM1 (637 ± 45 vs. 390 ± 16, pg/ml, *p* < 0.01) were significantly higher in COVID-19 compared to controls (Fig. 1B). Of the 38 CCs investigated, 7 CCs were significantly higher and 2 CCs were significantly lower in COVID-19 (Fig. 1C). The 7 CCs that were significantly higher were GRO (254 ± 29 vs. 79 ± 5.19, pg/ml, *p* < 0.01), IL10 (27 ± 5.7 vs. 7.4 ± 2.5, pg/ml, *p* < 0.01), sCD40L (144 ± 32 vs. 40 ± 6, pg/ml, *p* < 0.0001), IL1Ra(318 ± 131 vs. 68 ± 28, pg/ml, *p* < 0.01), IP10 (2952 ± 512 vs. 148 ± 20, pg/ml, *p* < 0.001), MCP1 (300 ± 34 vs. 154 ± 8, pg/ml, *p* < 0.05) and TNFa (10 ± 0.5 vs. 5 ± 0.4, pg/ml, *p* < 0.01). The two CCs that were significantly lower in the COVID-19 cohort were MDC (158 ± 12 vs. 231 ± 26, pg/ml, *p* < 0.05) and MIP1a (2.5 ± 0.4 vs. 4.2 ± 0.8, pg/ml, *p* < 0.05). The levels of all non-significant BIMs, CCs and EIMs in Control vs. COVID-19, male vs. female COVID-19 subjects and across severity are tabulated in Tables S1, S2 and S3 in the Additional file 1.

### The association with inflammatory cytokines and COVID-19

Bioinformatics (chord diagrams) analysis reveals distinct association profile between the COVID-19 and control cohort (Fig. 1D). Within the COVID-19 cohort, we observed high positive correlations between the BIM and inflammatory CCs indicative of a brain injury mechanism triggered by systemic inflammation. While IL6 levels were not significantly higher in the COVID-19 cohort, as previous studies have found they trended higher in men. In our cohort, we observed IL6 to highly correlate with other CCs and BIMs (Fig. 1D). Several inflammatory CCs and notably MCP1, IP10, IL6, IL1Ra, IL10 and TNFa had more associations (Fig. 1D, COVID-19) with the BIMs suggesting that these CCs are the inflammatory markers driving brain injury.

### Severity and outcomes

**Fig. 2:**
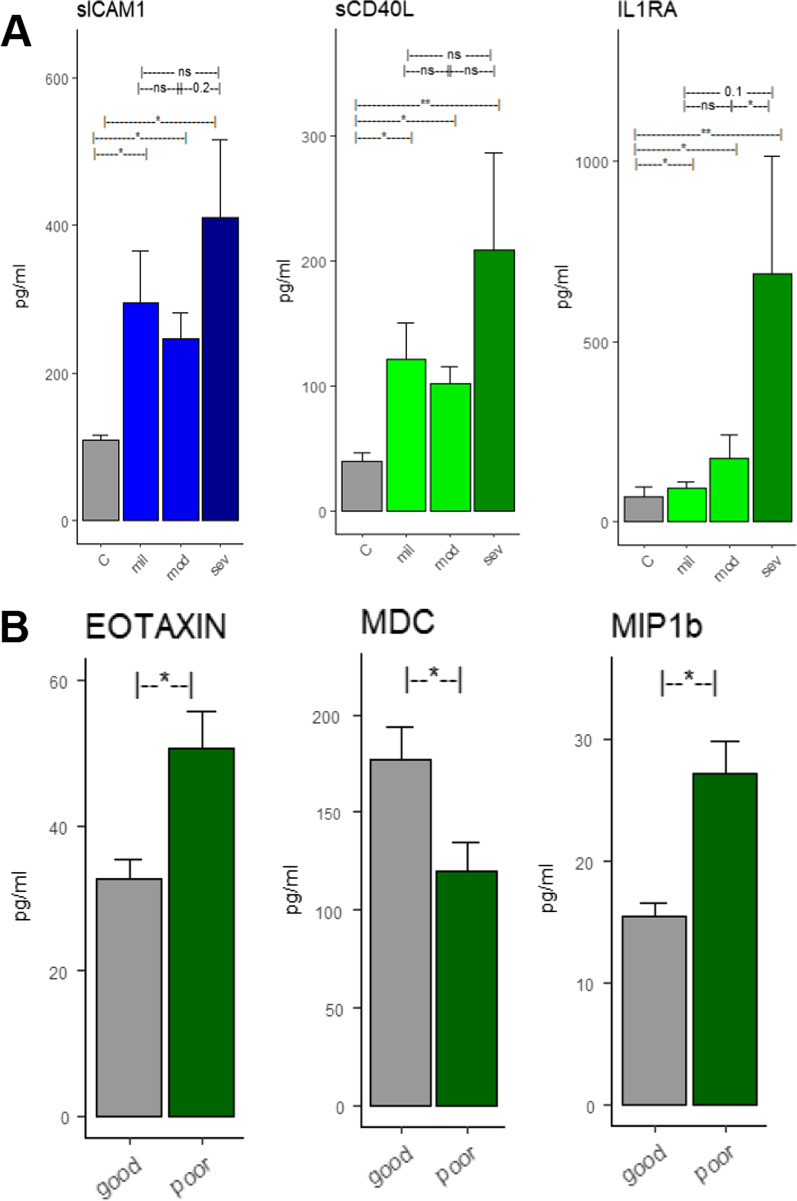
Differences across severity and clinical outcomes. Differences across severity and clinical outcomes. **A** Difference in SICAM1, sCD40L and IL1RA across controls, mild, moderate and severe COVID groups. **B** Difference in CC levels across functional outcomes at discharge. EOTAXIN, MDC and MIP1b were significantly different across the good and the poor functional outcome groups. However, when adjusted for age and sex, only MIP1b was independently associated with outcomes

We examined the levels of BIMs, EIMs and CCs across COVID-19 severity (mild, moderate and severe). The levels of BIM were not significantly different across severity. However, one EIM (sICAM1) and two CCs (SCD40L and IL1RA) showed a near-significant trend of elevation in high severity (Fig. 2A). None of the BIMs or EIMs were predictive of functional outcomes at discharge. Two CCs, Eotaxin and MIP1b were significantly higher (*p* < 0.05) in subjects who proceeded to have poor outcomes (Fig. 2B). When adjusted for age and sex, only MIP1b was found to be independently associated with functional outcomes. MDC was significantly (*p* < 0.05) lower in those who proceeded to have poor outcomes. However, when controlled for age and sex in a multivariate logistic regression model, only MIP1b was associated with functional outcomes (Fig. 2B).

### Sex differences

**Fig. 3:**
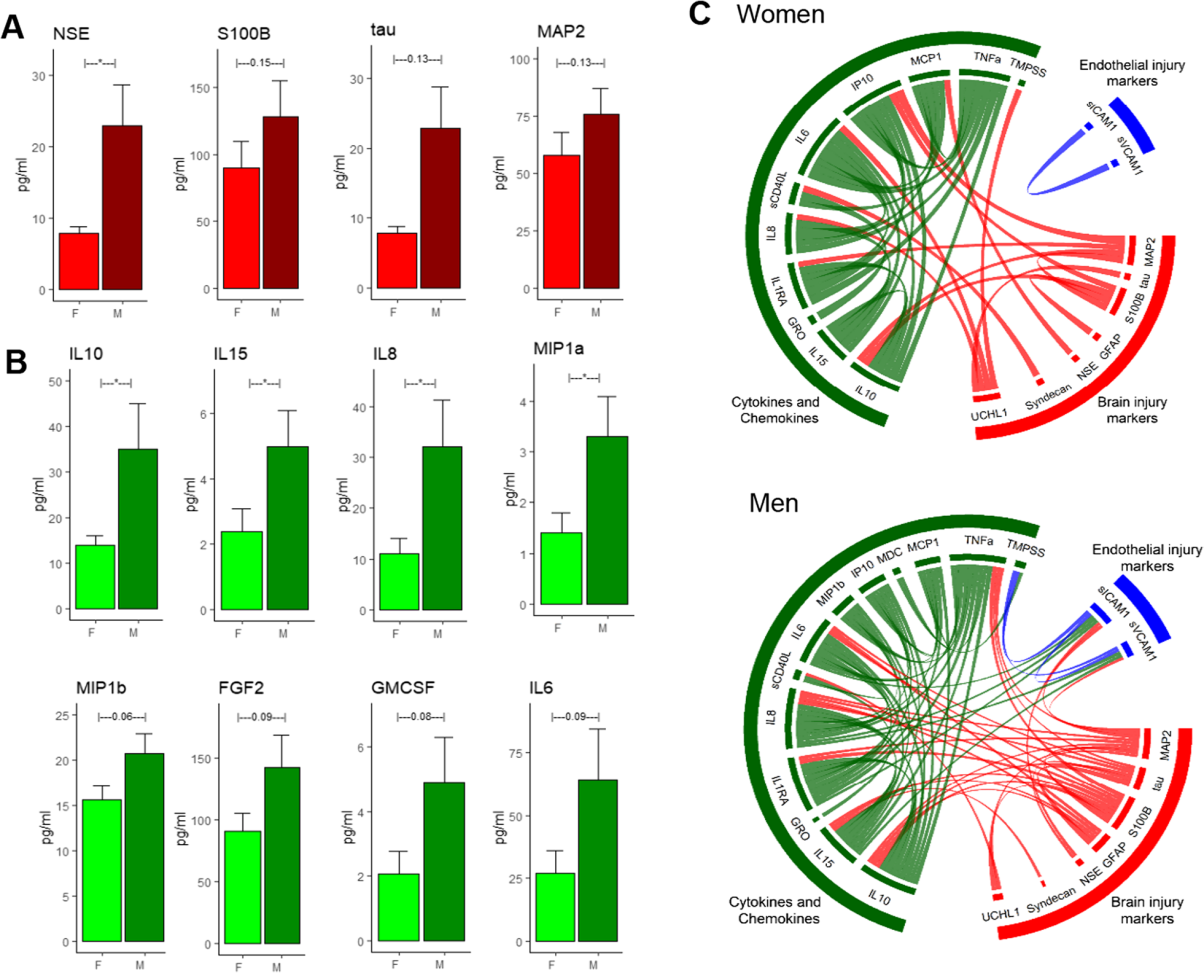
Sex difference in BIM and CC profile across COVID-19 subjects. **A** NSE was significantly (*p* < 0.05) higher after COVID-19. S100b, tau and MAP2 were higher but differences were not statistically significant **B** IL10, IL15, IL8 and MIP1a were significantly (*p* < 0.05) in males compared to females. MIP1b, FGF2, GM-CSF and IL6 indicated a non-significant trend of being elevated in males. **C** The BIM–EM–CC interactions reveal that males have a much robust interactions compared to females. The results indicate that there was a robust association between the inflammatory-brain injury markers in ‘males’ compared to ‘females’. Males had 75 significant (*p* < 0.05) associations between the markers compared to females who only had 39 significant associations indicating a higher overall inflammatory-mediated brain injury marker mechanisms in male compared to females. The similarity index (SI) between two matrices is 0.71, a *p*-value < 0.0001 using the random skewers method

Among the BIMs, only the levels of NSE was significantly higher in men compared to women (7.95 ± 0.9 vs. 22.9 ± 5.8, *p* = 0.01). BIMs including S100B, tau and MAP2 were higher in men; however, these differences were not statistically significant (Fig. 3A). The levels of the EIMs were not significantly different across men and women (data not shown). Four CCs were significantly higher in men compared to women: IL10 (35 ± 10.2 vs. 14.4 ± 2.3, *p* = 0.05), IL15 (5 ± 1.1 vs. 2.4 ± 0.6, *p* = 0.4), IL8 (32 ± 9.2 vs. 11.4 ± 3.06, *p* = 0.04) and MIP1a (3.3 ± 0.7 vs. 1.4 ± 0.4, *p* = 0.4). Four CCs including MIP1b, FGP2, GM-CSF and IL6 were near significantly (*p* < 0.1) higher in men compared to women (Fig. 3B). The BIM–EIM–CC interaction profile reveal stark differences between men than women (Fig. 3C). Men had a higher number of correlations between the BIM–EIM–CC and the correlation profile was significantly different compared to women (*p* < 0.05, Fisher’s test). There were more interactions between the CCs and BIMs suggestive of a higher degree of inflammation induced brain injury in men. Additionally, in women, the EIMs (sICAM1 and SVCAM1) were not significantly correlated with the CCs or BIMs. However, in men, the EIMS were highly correlated with CCs and BIMs (Fig. 3C).

The expression levels of all the proteins (BIMs, CCs and EIMs) that were not significant (*p* > 0.05) across the groups (i.e., across control vs. COVID-19, across sex and across severity) are presented (see Additional file 1: Tables S1, S2 and S3).

## Discussion

We investigated the profile of brain injury, endothelial injury and inflammatory markers after COVID-19 disease. We found that levels of BIMs, specifically MAP2, NSE and S100β were significantly elevated in the acute phase of COVID-19. Two EIMs, sICAM1 and sCAM-1, were elevated after COVID-19 infection with robust interactions with the BIMs and IL6. Additionally, a host of inflammatory markers was elevated after acute COVID-19 infection. Interestingly we found men expressed significantly higher BIM levels than women suggesting a strong sex-dependent biological mechanism underlying the interactions between systemic inflammation, endothelial damage and brain injury after COVID-19. Even among the proteins that were non-significant across the groups, there was a trend towards increased inflammatory and injury markers in COVID-19 subjects and in men compared to women.

The neuro-invasive and neurotropic nature of human coronaviruses (HCoV) are known, and they may contribute to both short- and long-term neurological disorders [9–11]. The neuroinvasion of HCoVs confirmed by the presence of viral RNA in the human CNS adds credence to this hypothesis [30]. Acute neurologic and psychiatric complications have been reported after COVID-19 hospitalizations [2]. A wide range of neurological manifestations including ischemic stroke, intracerebral hemorrhage, hallucinations, encephalopathy, anosmia and ageusia have been described after SARS-CoV-2 infection [3–8]. Hyperemic and edematous brain tissue and neuronal death have been reported in autopsy cases after COVID-19 [31]. However, it is unknown if BIM are elevated in patients without overt neurological disease.

To investigate the effects of COVID-19 on BIMs, we examined systemic levels of six previously validated BIMs—MAP2, NSE, GFAP, S100B, Syndecan-1 and UCHL1. We found that MAP2, NSE and S100B were higher after COVID-19 indicative of brain injury after COVID-19. MAP-2 is a dendritic injury marker that is elevated in both the acute and chronic stages of brain injury [32]. This protein is typically localized in the dendritic regions and is involved in the promotion of microtubule synthesis and cross-linking with other compartments of the cytoskeleton. It is elevated after a traumatic brain injury and associated with long-term outcomes [32]. NSE is an acidic protease present in neurons and neuroendocrine cells and is a useful indicator of neuronal damage [33]. S100B is a calcium-binding protein that is localized in astrocytic cytoplasm. It has been shown to be elevated systemically especially in traumatic brain injuries [34] and strokes [35]. Systemic levels of S100B are higher after traumatic brain injury and have been associated with poor long-term outcomes [36–38]. Increased levels of MAP2, NSE and S100B indicate neuronal and astrocytic injury after COVID-19 (Fig. 1A). Additionally, we found that BIMs (NSE, S100β, tau, MAP2) were higher in men compared to women with COVID-19. It is not clear whether the elevations in BIMs overall and specifically in men have clinical relevance. We deliberately excluded samples from subjects with overt neurologic injury (i.e., traumatic brain injury, ischemic stroke, or hemorrhagic stroke) associated with COVID-19 disease. The elevated BIMs suggest that there is CNS involvement in acute COVID-19 and the clinical ramifications of this for chronic outcomes requires investigation.

Several pro-inflammatory cytokines including IL10, MCP1, TNFa, MDC, GRO, sCD40L, IL1Ra and IP10 were higher after COVID-19 compared to controls consistent with findings from several recent studies [39–41]. We also found that EIMs (sICAM-1 and sVCAM-1) were higher after COVID-19 infection (Fig. 1B). Endothelial cells express the ACE-2 receptor—the primary receptor and main route in intracellular entry for SARS-CoV-2 [42]. The blood–brain barrier (BBB) via the endothelial tight junctions is critical in maintaining cerebral homeostasis and BBB breakdown is implicated in variety of neurological diseases [43]. The BBB is compromised in COVID-19 and the SARS-CoV-2 spike protein can cross the BBB into brain tissue [44, 45]. The endothelial dysfunction might be a direct result of the SARS-CoV-2 infection or due to a reaction to systemic inflammation triggered by the viral infection, or a combination of these factors. ICAM-1 is expressed in the vascular endothelium and is typically induced by IL-1b and TNFα [46]. It promotes leukocyte adhesion [47] and affects barrier function and vascular leakage [48]. sICAM-1 is the circulating form of ICAM-1 and has been identified as a candidate marker of vascular inflammation [49]. Increased levels of sICAM-1 have been observed in cardiovascular disease including myocarditis [50] and inflammatory cardiomyopathy [51]. In addition to increased levels of sICAM-1, we found that TNFα was also elevated after COVID-19. In our data, the simultaneous elevation of the pro-inflammatory TNFα and sICAM-1 is suggestive of an inflammatory-mediated vascular injury rather than a virally triggered vascular injury (Fig. 1). In fact, a recent NIH funded post-mortem study examining the brain tissue of deceased COVID-19 subjects reported extensive inflammation and microvascular blood vessel damage, but it is not clear how SARS-CoV-2 is involved in the brain injury and neurological outcome [52]. The findings of this study suggest that the microvascular blood vessel damage is likely caused by the inflammatory response due to the infection, rather than the virus itself.

Interestingly, the anti-inflammatory marker IL10 was elevated in COVID-19 subjects. The elevation of IL10 could simply be a response to elevations in pro-inflammatory processes after COVID-19 infection in addition to other inflammatory markers. Alternatively, this could indicate the activation of immunosuppression mechanisms, which has been implicated in poor outcomes in brain injury. Elevation in IL10 supports the idea that early immune suppression may play a detrimental role in brain injury. Perhaps a dysregulation of the immune process in addition to overwhelming inflammation is the driver of pathophysiological processes [53]. IP-10 is shown to be involved in both pro-inflammatory and regulatory mechanisms and IP-10 signaling is a known promoter of T-cell recruitment and activation [54]. Two pathways involving IP-10: the restraining of IP-10 production by CCL5 [55] and the inhibition of regulatory T-cell recruitment by IP-10 [54], have been previously implicated in controlling inflammatory response and in neuro protection [56, 57]. Another study has reported an increased level of IP10 and MCP1 as biomarkers after COVID-19 [58]. IP-10 can also inhibit endothelial recovery [59] and investigations in anti-IP10 antibody is underway to test to promote endothelial healing. A similar anti-IP10 antibody treatment has been suggested in COVID-19 patients with thrombotic events.

Neuronal injury after COVID-19 infection is documented and multiple mechanisms via of CNS damage including viral mediated injury, hyper-inflammation and tissue hypoxia due to respiratory failure have been purported. Direct CNS infection by SARS-CoV has been shown in humans and mice [11], and SARS-CoV-2 appears to have the potential to directly infect the brain and cerebral vasculature of humans. However, it is unclear whether the CNS damage is a result of the viral infection of due thrombotic microangiopathy and systemic hyper-inflammation. An alternate mechanism of neuronal injury includes CNS hypoxia due to respiratory failure. Our finding that severity of respiratory compromise did not correlate with BIM suggests the brain hypoxia is not the dominant cause for brain injury. On the other hand, there is increasing mechanistic evidence that neuronal injury is mediated primarily via hyper-inflammation and endothelial dysfunction. A study examining neuronal injury after COVID-19 patients reported the presence CNS damage likely due to endothelial leakage [60]. This post-mortem study used investigated MRI scans of olfactory bulbs and brainstems in 16 COVID-19 patients and reported the presences of hyperintensity and hypointensity regions—which indicate the presence of inflammation and bleeding, respectively. They reported the presence of thin endothelial vessels and the presence of fibrinogen—a marker of BBB leakage. The hyperintensity regions were surrounded by T cells and microglia and the hypointensity regions contained both clotted and leaky blood vessels but no immune response. Additionally, they reported no evidence of SARS-CoV-2 infection in the brain tissue suggesting that the CNS injury was not caused by the direct viral attached in the brain, but rather due to microvascular blood vessel damage as the body’s response to the virus. The authors also ruled out injury due to CNS hypoxia as the areas of damage were multifocal that are typically observed in strokes and other neuroinflammatory diseases. In another study, a SARS-CoV-2-mediated neuroinflammatory response was reported via investigating small cell clusters of early-activated macrophages in the olfactory epithelium [61]. An upregulation of (HLA)-DR on macrophages and increased levels of myeloid-driven inflammatory response (including an elevated levels of IL6, IL18, CCL2 and sICAM-1 in the CSF) were reported. Furthermore, histopathological investigations indicated micro-thrombosis and acute brain infarcts in 18% of the subjects.

To elucidate the relationships between the BIMs, EIMs and systemic CCs in our cohort of subjects, we employed a bioinformatics visualization tool called the chord diagram and overlaid the significant correlations. In the COVID-19 cohort, the systemic CCs IL6, IL1Ra, IL10, TNFa, MCP1 and IP10 had high associations with the BIMs. Among the BIMs, MAP2 and S100B had the most associations with the systemic CCs (Fig. 1D). Particularly, the pro-inflammatory cytokines TNFa, IP10, IL1Ra and IL6 were strongly correlated with the MAP2 and S100B which were absent in the control group (Fig. 1). The relationship between brain injury and excessive systemic inflammation has previously been reported in other acute diseases [62–64]. The systemic inflammatory signals are purported to affect brain regions via either the humoral/neural pathways (via a compromised BBB) or via the activation of the vagus nerve [65–67]. For instance, systemic levels of TNFa have been reported to mediate brain injury processes after TBI and stroke [68] and inflammation in astrocytes [69]. Experimental models have shown that the deficiency of TNFa results in less cortical tissue loss after brain injury [70]. IL6 has also been shown to mediate brain injury. The strong association between the BIMs and the pro-inflammatory IL6 and TNFa in our cohort suggests the presence of inflammation-mediated neuronal injury mechanisms. Besides specific associations, there were relatively higher correlations between the BIMs and CCs in the COVID-19 cohort compared to controls suggest inflammation induced brain injury in COVID-19. It is known that inflammatory diseases are associated with neuronal injury (as in the case of encephalitis after sepsis [64]). Though our study design is non-mechanistic and direct causative mechanisms cannot be inferred, it is plausible that our findings, taken together with previous studies, support the view that the extensive cytokine activation by COVID-19 infection is the driver of endothelial injury that leads to neuronal and astrocytic injury in COVID-19.

Interestingly, the levels of BIMs were not different across clinical severity or clinical outcomes. sICAM1 and two CCs (sCD40L and IL1Ra) were higher in severe grade subjects (Fig. 2A), but this difference was not significant. Three CCs (Eotaxin, MDC and MIP1b) were different across clinical outcomes (Fig. 2B). After adjusting for age and sex, only MIP1b was independently associated with clinical outcomes. The lack of differences in BIMs levels across severity or clinical outcomes is likely a reflection of the time-point at which the samples were drawn rather than a pathophysiological mechanism of the disease process itself. Subject severity (mild, moderate or severe) was based on the course of hospital events (like requirement of high-flow cannula for extended time-period, ventilator, etc.) and not based on the symptoms that were observed at admission. Since we only used the first time-point for this study (obtained at < 48 h after admission)—which is around the time of admission, it is likely that these samples reflect a protein profile from a cohort of homogenous severity subjects. Additionally, the sample size of this study is relatively low and these observations need to be confirmed in another cohort with a larger sample size.

The long-term neurological effects of COVID-19 are increasingly recognized [71]. As more COVID-19 patients are recovering from hospitalization, it is expected that many discharged patients will be affected with secondary complications and symptoms [72]. These symptoms are referred to as post-acute sequelae of COVID-19 or of SARS-CoV-2 (PASC) and include a host of medical symptoms that include functional, cognitive and psychiatric symptoms [73–75].

COVID-19 disproportionately affects men more severely compared to women. Preliminary studies have suggested that while the prevalence of infection is the same in men and women, men are more likely to be hospitalized, have a more severe disease course, and higher mortality than women [15]. We investigated differences in BIMs and CCs across men and women and we found that NSE was significantly elevated in men compared to women. Additionally, MAP2, tau and S100B showed trends towards elevated levels in men (Fig. 3A). Several CCs including IL15, IL8, MIP1b and IL10 were elevated in men compared to women (Fig. 3B) suggesting an increased inflammatory response in men. Men had a higher degree of inflammatory interactions (Fig. 3C) compared to women. Additionally, the interactions between the inflammatory markers (CCs) and the BIMs (red lines) were significantly higher in men indicating a higher degree of inflammation-mediated brain injury in men during the acute stages of COVID-19 (Fig. 3C). Interestingly, the interactions between the EIMs and the BIMs/CCs were also higher in men compared to women (Fig. 3C). This suggests that men likely experience a higher degree of inflammation-triggered endotheliopathy—an important factor to be considered when developing treatment plans. A number of reasons have been postulated for the differential response across sex to COVID-19. Some studies have shown that men have a higher level of ACE2 receptors, used for SARS-CoV-2 virus entry [76]. Others have postulated that the differences are due to differences in lifestyle factors and co-morbidities between men and women [76, 77]. Given our small sample size, we are not powered to determine if these differences in BIMs translate into worse neurologic outcomes. Our finding add to the studies that show men have a more robust humeral response to COVID-19 infection. We show that the inflammatory response is more tightly associated with endothelial disfunction and brain injury in men compared to women emphasize the sex-specific reactions to COVID-19. Despite the sex differences seen in COVID-19, treatment protocols and interventional studies have largely ignored these differences in favor of treatment regiments that are uniform across sexes. As we now transition into examining the long-term consequences of COVID-19, these sex differences in connections between inflammation, endothelial injury and brain injury need to be addressed for identification of successful therapeutics. Future studies investigating sex differences in the immune response to COVID-19 are required.

### Limitations

Our study has several limitations. First, we included samples only from the first-available time-point after hospitalization. Typically, the measurements from the first-available time-point are a reflection of the patient’s status during the earlier course of disease progress. However, this time-point is also the most accurate, as in our hospital all COVID-19 patients were administered dexamethasone, which could have a confounding effect on the measurements. Second, we have not related the observed BIMs with any long-term clinical outcomes. Third, out study has a relatively low sample size. We are underpowered to detect the effect of any co-morbidities on the neuronal injury. Finally, this is an observational study and the study design only allows for correlational associations. Further studies are required to establish direct causative mechanisms.

## Conclusion

Our findings suggest that immediately after COVID-19 hospitalization, BIMs were higher than matched controls. The levels of EIMs and CCs were also higher. The CCs were highly correlated with BIMs and EIMs, suggesting inflammatory-driven endothelial dysfunction and brain injury in the acute phase of COVID-19. Further studies are required to clarify the exact mechanisms how COVID-19 induces inflammation and brain injury, sex-specific differences and whether these early increase in injury markers are associated with long-term neurologic outcomes.

## Supplementary Information

**Additional file 1.** Protein differences (non-significant) in COVID-19 vs Controls, sex (within COVID-19 subjects) and across COVID-19 severity.

## Data Availability

All data generated and analyzed during this study are available from the corresponding author on reasonable request.

## Abbreviations

BIM: Brain injury markers
CC: Cytokines/chemokines
EIM: Endothelial injury markers
mRS: Modified Rankin Score
BBB: Blood–brain barrier
SARS-CoV: Severe acute respiratory syndrome-associated coronavirus
UCHL-1: Ubiquitin carboxy-terminal hydrolase L1
MAP2: Microtubule-associated protein 2
GFAP: Glial fibrillary acidic protein
NSE: Neuron-specific enolase
sICAM1: Soluble intercellular adhesion molecule-1
sVCAM1: Soluble vascular cell adhesion molecule 1
