# Supplementary material for: Brain injury, endothelial injury and inflammatory markers are elevated and express sex-specific alterations after COVID-19
Source: J Neuroinflammation. 2021 Nov 27;18:277. doi: 10.1186/s12974-021-02323-8 (PMC8627162; doi:10.1186/s12974-021-02323-8)
Supplement: Supplementary file 1 — Additional file 1. Protein differences (non-significant) in COVID-19 vs Controls, sex (within COVID-19 subjects) and across COVID-19 severity. [file 12974_2021_2323_MOESM1_ESM.docx]

**Table S1**: Protein differences (non-significant) in COVID-19 vs Controls

| **Proteins** | **Control** | **COVID-19** | ***p-value*** |
| --- | --- | --- | --- |
| **Cytokines/Chemokines** | **mean(se)** | **mean(se)** |  |
| FGF2 | 119(23) | 122(15) | 1 |
| EOTAXIN | 42.3(4.38) | 36(2.5) | 0.27 |
| TGFa | 4(1.45) | 3.9(1) | 0.5 |
| GCSF | 35.4(10.5) | 48(7.6) | 0.288 |
| Flt3L | 7.9(2.1) | 11.3(2.4) | 0.7 |
| GMCSF | 12(6.24) | 3.8(0.8) | 0.9 |
| Fractalkine | 112(25) | 135(18) | 0.5 |
| IFNa2 | 12.7(3.9) | 17.8(2.7) | 0.2 |
| IFNG | 8.5(2.9) | 11.2(3.3) | 0.36 |
| MCP3 | 158(38) | 96(18) | 0.32 |
| IL12P40 | 20.7(7.8) | 18.9(4.28) | 0.35 |
| IL12P70 | 3.46(1.68) | 16.2(10.9) | 0.35 |
| IL13 | 32.6(11.5) | 16.2(4.8) | 0.31 |
| IL15 | 3.77(1.4) | 3.96(0.65) | 0.36 |
| IL17A | 7.18(4.13) | 2.15(0.77) | 0.62 |
| IL1a | 77.3(32.7) | 55.44(15.5) | 0.59 |
| IL9 | 30.9(11.12) | 16.66(4.8) | 0.65 |
| IL1b | 1.94(0.58) | 1.99(0.399) | 0.72 |
| IL2 | 0.98(0.204) | 1.19(0.18) | 0.58 |
| IL3 | 0.5(0.03) | 0.55(0.02) | 0.399 |
| IL4 | 768(315) | 396(122) | 0.13 |
| IL5 | 13.28(4.27) | 5.22(1.32) | 0.59 |
| IL6 | 60.4(19.8) | 49(11.5) | 0.71 |
| IL7 | 2.05(0.97) | 3.09(1.02) | 0.91 |
| IL8 | 20.6(6.93) | 23.68(5.19) | 0.39 |
| MIP1b | 14.1(1.33) | 18.6(1.35) | 0.31 |
| TNFb | 202(76) | 114(37.8) | 0.17 |
| VEGF | 20.9(10.7) | 30.5(10.6) | 0.93 |
| **Brain Injury Markers** |  |  |  |
| GFAP | 2.68(0.9) | 3.91(1.22) | 0.7 |
| UCHL1 | 93(20) | 62(10) | 0.68 |
| Syndecan |  | 33(6.7) | 0.26 |

All values in pg/ml

**Table S2**: Protein differences (non-significant) across Sex in COVID-19 subjects

| **Proteins** | **Female** | **Male** | ***p-value*** |
| --- | --- | --- | --- |
| **Cytokines/Chemokines** | **mean(se)** | **mean(se)** |  |
| EGF | 8.48(2.16) | 12(2.8) | 0.68 |
| FGF2 | 91(15) | 143(26) | 0.3 |
| EOTAXIN | 32(2.8) | 39(4.2) | 0.46 |
| TGFa | 2.11(0.68) | 5.07(1.95) | 0.18 |
| GCSF | 35.8(8.5) | 57.1(12.9) | 0.76 |
| Flt3L | 8.2(3) | 13.5(3.9) | 0.3 |
| GMCSF | 2.07(0.7 | 4.9(1.4) | 0.37 |
| Fractalkine | 117(25) | 147(30) | 0.8 |
| IFNa2 | 13.9(1.2) | 20.5(4.37) | 0.37 |
| IFNG | 5.94(1.4) | 14.9(6.14) | 0.8 |
| MCP3 | 66(22) | 116(30) | 0.58 |
| IL12P40 | 12.4(5.26) | 22(7.2) | 0.37 |
| IL12P70 | 2.27(0.79) | 25(20) | 0.27 |
| IL13 | 8(4.63) | 21(8.3) | 0.62 |
| IL15 | 2.4(0.6) | 5(1.1) | 0.07 |
| IL17A | 1.32(0.53) | 2.72(1.4) | 0.49 |
| IL1a | 30.2(11) | 73(28) | 0.76 |
| IL9 | 9.6(5.2) | 21.5(8.3) | 0.67 |
| IL1b | 1.36(0.32) | 2.4(0.7) | 0.8 |
| IL2 | 0.85(0.17) | 1.4(0.32) | 0.58 |
| IL3 | 0.53(0.03) | 0.56(0.04) | 0.5 |
| IL4 | 216(129) | 520(2011) | 0.98 |
| IL5 | 3.09(1.39) | 6.6(2.28) | 0.28 |
| IL6 | 27(9) | 64(20) | 0.47 |
| IL7 | 1.2(0.74) | 4.3(1.8) | 0.09 |
| IL8 | 11.4(3.) | 32(9.2) | 0.11 |
| MIP1b | 15.6(1.6) | 20(2.2) | 0.14 |
| TNFb | 49(26) | 158(68) | 0.73 |
| VEGF | 11.7(7.7) | 43(19) | 0.3 |
| **Brain Injury Markers** |  |  |  |
| GFAP | 3.28(1.4) | 4.34(2.0) | 0.77 |
| UCHL1 | 40(15) | 77(16.3) | 0.17 |
| Syndecan | 40(14.6) | 28(8) | 0.75 |
| **Endothelial Injury Markers** |  |  |  |
| sICAM1 | 281(66) | 342(84) | 0.61 |
| sVCAM1 | 647(80) | 629(64) | 0.71 |

All values in pg/ml

**Table S3**: Protein differences (non-significant) across severity in COVID-19 subjects

| **Proteins** | **control** | **mild** | **moderate** | **severe** |
| --- | --- | --- | --- | --- |
| **Cytokines/Chemokines** |  |  |  |  |
| EGF | 14.3(2.9) | 8.5(2.6) | 11(3.5) | 12.2(3.7) |
| FGF2 | 119(23) | 94(17) | 124(17) | 147(30) |
| TGFa | 4(1.45) | 2.9(1) | 2.5(0.69) | 6.12(2.4) |
| GCSF | 35.4(10.5) | 42(9.8) | 34(9.3) | 68(13.5) |
| Flt3L | 7.9(2.1) | 10.9(3.2) | 9.5(2.5) | 13.6(4.6) |
| GMCSF | 12(6.24) | 2.5(0.7) | 3.5(0.9) | 5.3(1.71) |
| Fractalkine | 112(25) | 118.3(25) | 138(22) | 149(34) |
| IFNa2 | 12.7(3.9) | 16(4) | 17(3.5) | 19(4.8) |
| IFNG | 8.5(2.9) | 8.4(2) | 8.7(2.6) | 16.5(7.8) |
| GRO | 78(5.4) | 235(29) | 238(27) | 287(63) |
| IL10 | 7.6(2.6) | 21.6(3.5) | 19(3.5) | 39(13.5) |
| MCP3 | 158(38) | 72(20) | 115(27) | 100(32) |
| IL12P40 | 20.7(7.8) | 16(5.4) | 19(5.7) | 20(7.3) |
| IL12P70 | 3.46(1.68) | 2.5(0.8) | 39(27) | 6.5(2.4) |
| IL13 | 32.6(11.5) | 10.3(4.8) | 19.2(6.6) | 19(8.7) |
| IL15 | 3.77(1.4) | 3.6(0.8) | 3.6(0.9) | 4.6(1) |
| IL17A | 7.18(4.13) | 1.1(0.2) | 1.7(0.5) | 3.6(1.8) |
| IL1a | 77.3(32.7) | 36(13) | 58.8(16.9) | 71(32.3) |
| IL9 | 30.9(11.12) | 9.6(4.5) | 19.4(6.3) | 21(9.2) |
| IL1b | 1.94(0.58) | 1.3(0.3) | 2.2(0.5) | 2.4(0.8) |
| IL2 | 0.98(0.204) | 1.03(0.2) | 1.2(0.2) | 1.4(0.36) |
| IL3 | 0.5(0.03) | 0.48(0.02) | 0.53(0.02) | 0.64(0.05) |
| IL4 | 768(315) | 241(110) | 451(160) | 496(236) |
| IL5 | 13.28(4.27) | 3.63(1.44) | 5.8(1.7) | 6.14(2.4) |
| IL6 | 60.4(19.8) | 29(9) | 53(15) | 64(22) |
| IL7 | 2.05(0.97) | 1.9(0.7) | 2.4(1) | 4.9(2.2) |
| IL8 | 20.6(6.93) | 14.7(3.2) | 19.6(4.6) | 36(11) |
| IP10 | 156(19) | 2946(663) | 3687(1027) | 2223(323) |
| MCP1 | 159(7) | 270(31) | 241(26) | 389(73) |
| MIP1a | 4.4(0.8) | 2.7(0.8) | 2.1(0.48) | 2.7(0.68) |
| TNFa | 5.2(0.4) | 9.5(0.8) | 9.4(0.7) | 10.7(0.8) |
| TNFb | 202(76) | 54(23) | 121(49) | 166(76) |
| VEGF | 20.9(10.7) | 19.9(8.4) | 21(9.9) | 50(22.8) |
| **Brain Injury Markers** |  |  |  |  |
| NSE | 5.1(0.5) | 17(5.6) | 18(4.7) | 15(3.4) |
| S100B | 45(6.8) | 120(19) | 60(5.6) | 156(34) |
| MAP2 | 25(3.9) | 69(8) | 62(8.7) | 74(14) |
| GFAP | 2.68(0.9) | 1.7(0.8) | 4.7(1.7) | 5.3(2.3) |
| UCHL1 | 31(11) | 19(4.4) | 28(8) | 41(13) |
| Syndecan | 19.6(6.2) | 43(11) | 28(11.2) | 28(5.2) |
| **Endothelial Injury Markers** |  |  |  |  |
| sICAM1 | 108(7) | 295(66) | 246(34) | 410(101) |
| sVCAM1 | **390(15)** | 634(59) | 633(67) | 642(67) |
